# Three-dimensional reconstruction of laryngeal cancer with whole organ serial immunohistochemical sections

**DOI:** 10.1038/s41598-020-76081-7

**Published:** 2020-11-03

**Authors:** Jun Tian, Bo Qian, Sanmei Zhang, Rui Guo, Hui Zhang, J.-P. Jeannon, Rongxiu Jin, Xiang Feng, Yangni Zhan, Jie Liu, Pengfei He, Jue Guo, Le Li, Yue Jia, Fuhui Huang, Binquan Wang

**Affiliations:** 1 Department of Otolaryngology, Head & Neck Surgery, Beijing Friendship Hospital, Capital Medical University, Beijing, China; 2 Department of General Surgery, The General Hospital of Taiyuan Iron & Steel Company, Taiyuan, China; 3 Medical Department of Medical Insurance, Beijing Friendship Hospital, Capital Medical University, Beijing, China; 4 Imaging Department, Shanxi Medical University, Taiyuan, China; 5 Department of Otolaryngology, Head & Neck Surgery, Guy’s & St Thomas NHS Hospital, London, UK; 6 Department of Nursing, Peking University Shenzhen Hospital, Shenzhen, China; 7 Department of Otolaryngology, Head & Neck Surgery, the First Hospital of Shanxi Medical University, No. 85, Jiefang South Road, Taiyuan, 030001 China; 8 Department of Head and Neck Surgery, Shanxi Provincial Cancer Hospital, Taiyuan, China

**Keywords:** Imaging, Immunological techniques, Microscopy, Cancer imaging

## Abstract

Three-dimensional (3D) image reconstruction of tumors based on serial histological sectioning is one of the most powerful methods for accurate high-resolution visualization of tumor structures. However, 3D histological reconstruction of whole tumor has not yet been achieved. We established a high-resolution 3D model of molecular marked whole laryngeal cancer by optimizing the currently available techniques. A series of 5,388 HE stained or immunohistochemically stained whole light microscopic images (200 ×) were acquired (15.61 TB).The data set of block-face images (96.2 GB) was also captured. Direct volume rendering of serial 6.25 × light microscopy images did not demonstrate the major characteristics of the laryngeal cancer as expected. Based on fusion of two datasets, the accurate boundary of laryngeal tumor bulk was visualized in an anatomically realistic context. In the regions of interest, micro tumor structure, budding, cell proliferation and tumor lymph vessels were well represented in 3D after segmentation, which highlighted the advantages of 3D reconstruction of light microscopy images. In conclusion, generating 3D digital histopathological images of a whole solid tumor based on current technology is feasible. However, data mining strategy should be developed for complete utilization of the large amount of data generated.

## Introduction

Cancer cells grow in all three dimensions. Ideally, understanding and evaluating the behavior and characteristics of solid cancer tissues in terms of oncology should include the information of the whole tumor in all the three dimensions, which is crucial for surgical planning and adjuvant therapy.

In clinical practice, magnetic resonance imaging (MRI) and computed tomography (CT) have significantly improved and have become more convenient for 3D reconstruction. However, they do not yield a satisfactory resolution to distinguish the tumor mass from normal tissue. Several studies have reported 3D models of tumor architecture based on reconstruction of light microscopic image sections^1–5^, which show promise in cancer research. However, ultrahigh-resolution 3D reconstruction of the entire bulk of a tumor is not yet to be achieved.

For any project, the preparation of whole-organ sections and data processing aimed at reconstructing a whole organ at the centimeter scale from microscopic sections is a challenging task. The difficulties encountered by researchers while building ultrahigh-resolution 3D models, from a mouse brain to a human brain, based on serial histological sections^6–8^, include high expenditure, time consumption, risk of sample loss, impairment, uneven dyeing, distortion during histological processing, digitalization possibilities, paucity of imaging programs designed for 3D light microscopy and current computational ability. These difficulties have not been overcome successfully.

Here, we obtained the dataset of an entire laryngeal cancer at a nearly subcellular resolution based on true serial sections. Using the dataset, we established 3D models with different resolutions to highlight the clinical application in terms of detailed anatomical relationships, tumor heterogeneity, proliferation and the cancer host interface. We took advantage of recent progress in laboratory techniques of whole-organ sectioning, whole-slide imaging systems, and improved light microscopic image reconstruction. Laryngeal cancer was selected because studies of whole-organ sections of the larynx have contributed most to the evolution of laryngeal cancer treatment^9–14^ and laryngeal cancer is unique because of its complicated spatial structure and multiple tissue types. In this study, we confirmed the possibilities and significance of 3D reconstruction of whole solid tumors at a centimeter level with an excellent resolution. Thus, more attention should be paid to optimize this powerful tool for cancer research.

## Results

A series of 6735 consecutive block-face images were acquired, the total volume of which was 96.2 GB. A total of 5800 histological sections were obtained at 58 mm thickness and were serially stained. An additional 16 sections were stained as a blank control outside of the series. Subsequently, the lower part of the block (nearly 9.35 mm high) was not stained because of the presence of tracheal rings beyond the larynx. Hence, the final numbers of each type of section were as follows: 1347 (HE), 1347 (Ki-67), 1347 (CK), and 1,347 (D2-40). After digitization at 200 ×, the volume of each primary dataset was 4.16, 4.00, 4.01, and 3.44 TB (maximally 13,000 × 11,000 pixels in size). The uninterrupted data acquisition time was nearly 2000 h.

### 3D reconstruction of the whole larynx from a macro perspective

**Figure 1:**
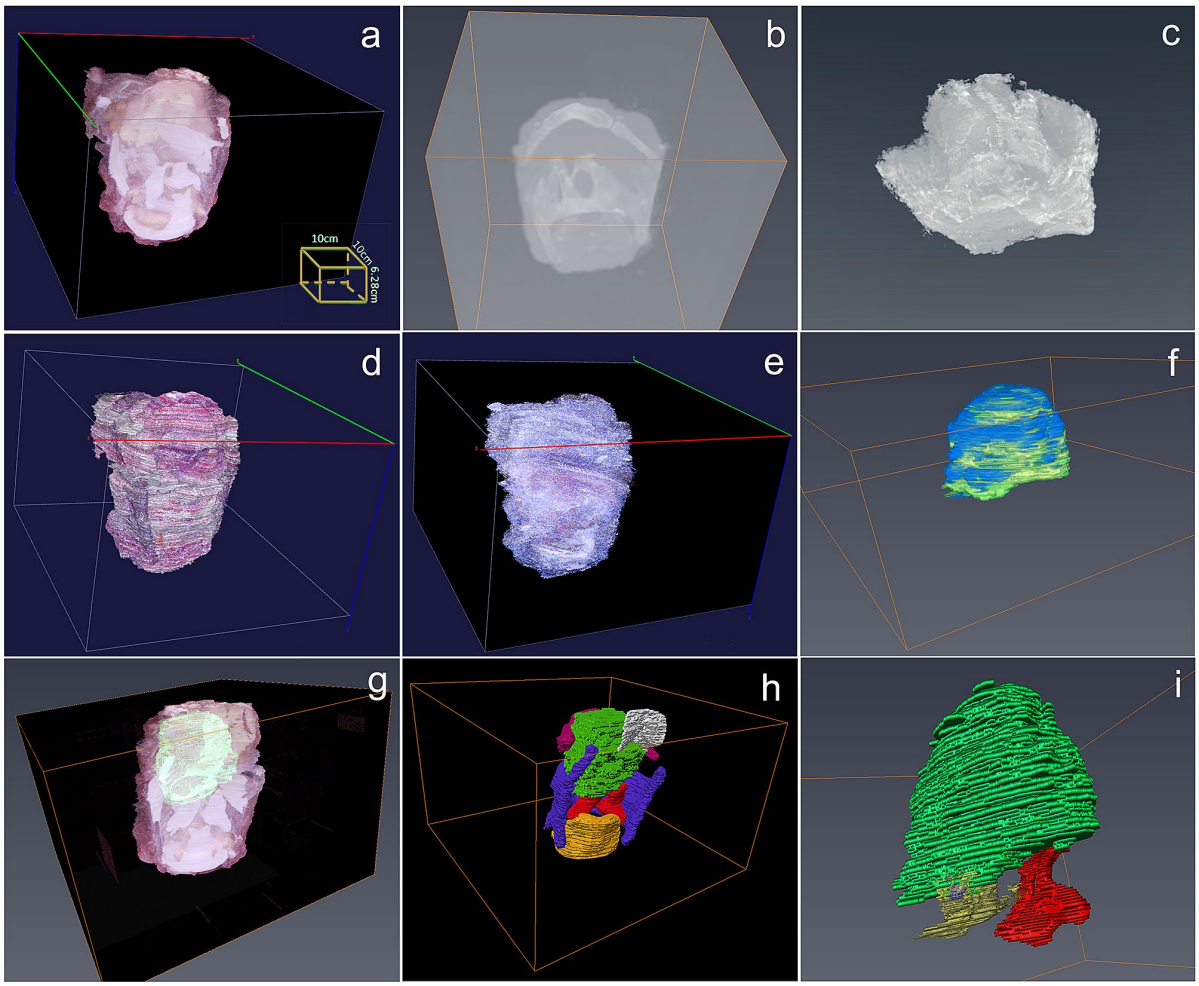
3D models and exploration of laryngeal cancer from a macro perspective. (**a**) 3D model of tumor bulk and framework of larynx without segmentation based on block surface image data; (**b**) 3D model based on CT scan without segmentation; (**c**) 3D model based on MRI scan without segmentation; (**d**) 3D model based on 6.25 × light microscopy images stained by HE without segmentation (Using alpha-blending projection method); (**e**) 3D model based on 6.25 × light microscopy images stained by pan-cytokeratin without segmentation (Using maximum intensity projection method); (**f**) Two kinds of boundaries of the tumor bulk identified from block surface images (Blue) and microscopic images (Green) shown in one 3D model; (**g**) Relatively accurate outline of the tumor bulk based on pathology (Green) was mapped to a 3D model based on block surface photographs without segmentation; (**h**) Relatively accurate outline of the tumor bulk based on pathology (Green) was mapped to a 3D model based on cross-sectional photographs after segmentation of epiglottis(White), hyoid bone(Pink), thyroid cartilage(Purple), arytenoid joint(Yellow and red); (**i**) Location of a small exophytic part of tumor(Purple) through the basement membrane of the ventricle (Yellow) with most of the mucosa of the ventricle remaining intact. (Vaa3D v3.544 used to generate Figure (**a**, **d**, **e**), https://alleninstitute.org; Amira 6.0 used to generate Figure (**b**, **c**, **f**, **g**, **h**, **i**), https://www.thermofisher.com).

Volume rendering of the whole serial block surface images provided valuable information about the 3D shape of the tumor bulk and framework of the larynx without segmentation (Fig. 1a, Supplementary Video 1), despite poor visualization of the bone and ossified cartilage. In contrast, 3D model based on CT and MRI without segmentation did not show the bulk of the tumor (Fig. 1b,c). It was hard to find an appropriate reconstruction algorithm to build a 3D model to demonstrate the major characteristics of the laryngeal cancer based on all 6.25 × light microscopy images, HE stained (Fig. 1d) and IHC stained (Fig. 1e) directly.

Two different boundaries of the tumor bulk, identified from the block surface photographs and microscopic images, can be fused in one 3D model (Fig. 1f).These were similar in most regions in macroscopic view except sites with an extensive host response at the inferior part. The accurately identified margin of the tumor bulk based on pathology was mapped to the 3D model, based on block surface photographs with or without segmentation of laryngeal frame (Fig. 1g,h, Supplementary Video 2, 3). It manifest the accurate boundary of tumor bulk in a real spatial context and promote understanding of the relationship between the tumor and the adjacent anatomical landmarks, such as epiglottis, hyoid bone, thyroid cartilage, ventricle, vocal cords, and arytenoid joint. The laryngeal ventricle could be identified in the pathological sections but not in block surface photographs because it was being severely compressed by the tumor. In fusion mode, the ventricle was well demonstrated in a stereogram. The location of a small exophytic part of the tumor, through the basement membrane of the ventricle, was marked in green in the model, even though most of the mucosa of the ventricle was intact (Fig. 1i). The front of the tumor was close to the arytenoid joint but had not invaded into it, which was easily presented in the spatial models (Fig. 1i, Supplementary Video 4) rather than 2D sections. Some tumor clusters appeared like skip lesions in 2D sections, while they appeared to be connected with the major tumor bulk in the 3D reconstruction model.

### 3D reconstructions of regions of interest (ROI) based on high-powered microscopy sections

**Figure 2:**
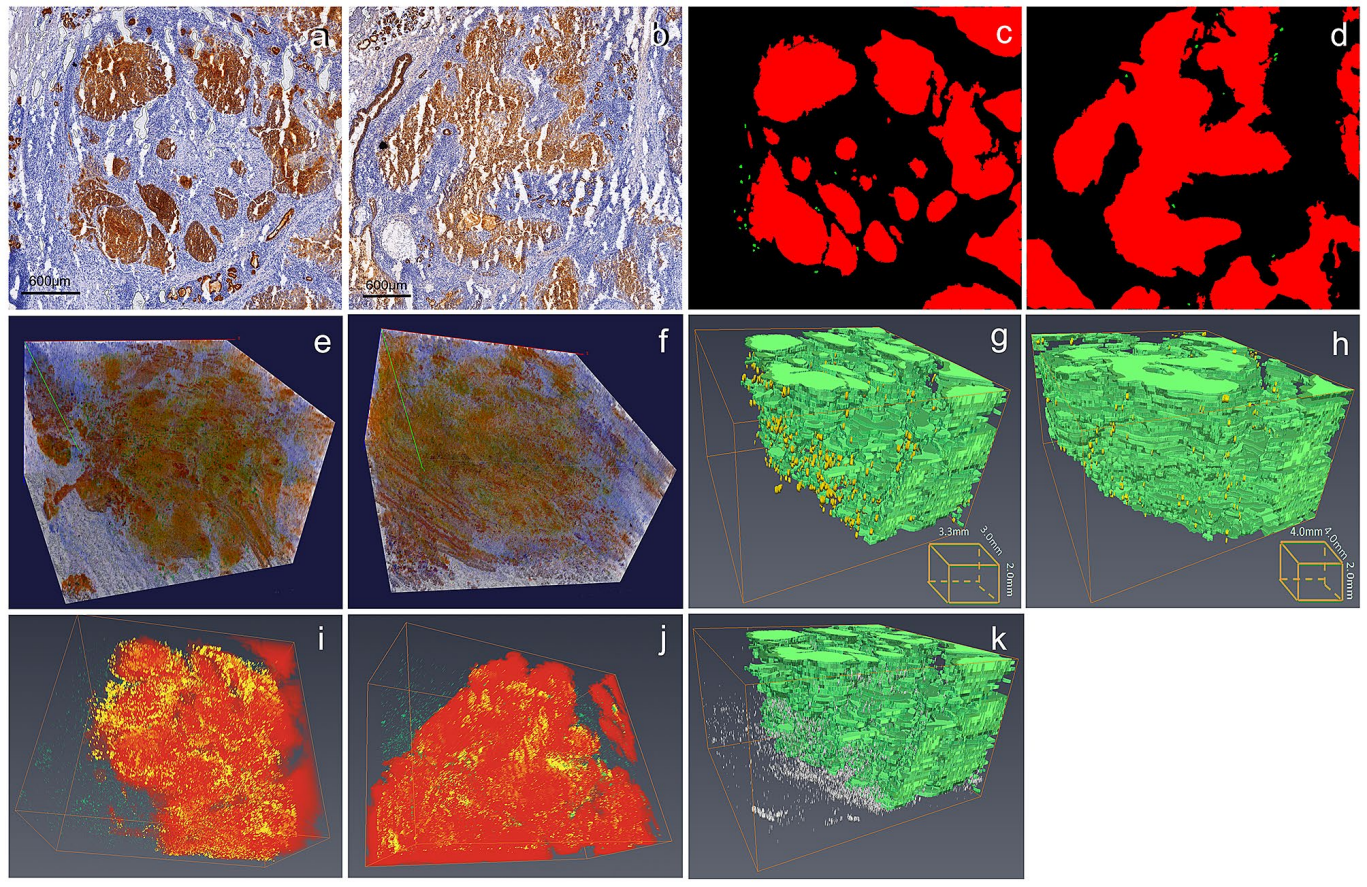
3D models and exploration of laryngeal cancer of two regions of interest based on high-powered microscopic sections from a micro perspective. (**a**, **b**) 2D section of two regions of interest based on high-powered microscopic sections stained by pan-CK (Round-shaped and palisading-shaped of tumor aggregates around the ventricle); (**c**, **d**) The segmentation and identification of tumor boundaries and buddings in 2D images of two regions of interest (buddings marked in green and tumors marked in red); (**e**, **f**) Direct 3D reconstructions of two regions of interest based on high-powered microscopic sections stained by pan-CK minimum using minimum intensity projection mode without segmentation (round-shaped and palisading-shaped of tumor aggregates around the ventricle), in which the tumor clusters are demonstrated in true color; (**g**, **h**) 3D model of regions of interest based on high-powered microscopic sections stained by pan-CK after segmentation (round-shaped and palisading-shaped of tumor aggregates around the ventricle, buddings marked in yellow and tumors marked in green); (**i**, **j**) 3D model of tumors marked by Ki-67 to demonstrate the spatial diversity of the proliferative activity of tumor cells (Ki-67 positive tumor cells marked with yellow, Ki-67 positive cells in stroma marked with green); (**k**) Model of lymphatic vessels in the intratumoral stroma after segmentation (lymphatic vessels marked in white and tumors marked in green). (Vaa3D v3.544 used to generate Figure (**c**, **d**), https://alleninstitute.org; Amira 6.0 used to generate Figure (**e**, **f**, **g**, **h**, **i**), https://www.thermofisher.com).

Around the ventricle, there existed two different areas in the 2D section. In one area, the tumor nests presented well round-shape (Fig. 2a). In the other, tumor aggregates showed fascicular or palisading shape (Fig. 2b).These two areas were chosen as ROI for high resolution 3D reconstruction (4.0 × 4.0 mm). The process of segmentation and identification of tumor boundaries and buddings in 2D images were showed in Fig. 2c and d. Before segmentation, the direct 3D reconstructions based on HE staining light microscopy did not provide valuable information as expected. After pan-cytokeratin staining, the tumor clusters were demonstrated directly in 3D exploration (Fig. 2e,f). After segmentation, round tumor nests in 2D sections did not appear spherical in three dimensions as expected (Fig. 2g), which suggest that the 2D shapes were more likely to represent a cross section of the tumor cord. Some small isolated round nests in 2D sections were proved to be protuberances originating from a relatively large tumor cell aggregate in 3D. In the ROI of the fascicular shape, the tumor cord connected more closely, like branches without an obvious trunk in 3D (Fig. 2h). Tumor budding was observed both at the tumor–host interface and in the intratumor stromal tissues in the two ROI (Fig. 2g,h, Supplementary Video 5, 6). Some identifications of tumor budding in 2D sections were found incorrect because they were found to be interconnected or connected to the main tumor branches when reconstructed in 3D. After incubation with Ki-67 antibody, cells located in the tumor superficial area had higher proliferative activity than those in the center, which was clearly shown in the 3D model (Fig. 2i,j).

In this study, any discrete D2-40-positive structure in the tumor stroma, regardless of the presence of lumen, was regarded as one lymphatic vessel and was three-dimensionally reconstructed in combination with budding (Fig. 2k). They were sparse in the stroma around the tumor nests, and did not display the lumen in 3D, the reason for which could be the collapse of intratumor lymphatic vessels during tissue processing.

### Preliminary exploration of tumor characteristics in the 3D model reconstructed from whole microscopic images using Vaa3D

After the precise alignment of 60 whole sections (50 ×) and the conversion of substantial image data in Vaa3D, multiresolution pyramid images were created, with a size of 120 GB. The Terafly moduleVaa3D provided a powerful tool for exploring whole-mount sections of human larynx at different scales in three dimensions (Supplementary Video 7).

## Discussion

In this study, we provide a feasible method to establish a high-resolution 3D model of entire molecular marked solid tumor by utilizing multiple currently available techniques, including whole-organ sectioning, molecular labeling, block-face image capturing, digital pathology panoramic scanning and 3D software for massive-scale (tera-voxels) image stacks. In our study, volume rendering of serial block surface images provided valuable information than models based on HE, IHC, MRI and CT. According to this approach, we visualized a whole laryngeal cancer bulk in an anatomically realistic context. Additionally, volume rendering of ROI based on IHC is better to describe the tumor in detail. In Tera Pyramid mode, we can explore local and global 3D views of tumors at different scales. This demonstrates the promising application of the tool in representing true 3D architecture of tumor-host interface, tumor budding, proliferating tumor cells and tumor lymph vessels.

Some studies^1–5,15–17^ have provided valuable 3D information on microscopic morphology or gene expression of different types of tumors, by reconstruction of a part of the tumor tissues from 75 to 500 serial slices using different systems. The entire tumor is hard to reconstruct completely at high resolution owing to a number of difficulties, such as, size limitations of slide scanner and microtome, poor uniformity/quality of serial whole-organ sectioning and staining, the time-consuming nature of the procedure, computer constraints, and limited software processing capability. Recently, researchers have succeeded in creating ultra-resolution 3D models of larger normal organs using different systems, ranging from mouse brain to human brain in size. Amunts et al.^6^ created an ultrahigh-resolution 3D model of a human brain named “BigBrain” based on the reconstruction of 7404 histological sections. The image was captured by a high-resolution flat-bed scanner, and the total volume of this dataset was 1 TB at a near-cellular resolution of 20 µm. Anan et al.^8^ obtained a 3D structural dataset of a Golgi-stained whole mouse brain at the neurite level using a micro-optical sectioning tomography (MOST) system, which can provide centimeter-scale tomography of centimeter-sized tissues and performs imaging and sectioning simultaneously. The total amount of uncompressed volume data exceeded 8 TB, with a voxel size of 0.33 × 0.33 × 1.0 μm, covering 15,380 coronal sections. Gao et al.^7^ achieved new 3D imaging of densely labeled mouse cortex and the entire Drosophila brain, which increased the resolution to nanometer-level over near-millimeter-level dimensions relying on the combination of expansion microscopy and lattice light-sheet microscopy. Until now, there is no ideal solution for high-throughput processing and ultrahigh-resolution 3D reconstructions of the entire bulk of a tumor are yet to be achieved, for each algorithm along with their advantages and disadvantages. In this study, we tried a commonly used process and obtained a dataset of 15.61 TB for the whole laryngeal cancer at a resolution of 0.47 × 0.47 × 10 μm. The advantages of this system were easy molecular labeling, accessible desktop devices and software. However, a large computerized cryomacrotome was required, which is crucial for extending its use to clinical practice.

Once the model was established, we evaluated if the model was worthwhile and provided some clues for optimization in the future. Our model verified that only whole 3D microscopic reconstruction could represent true 3D boundaries of a solid tumor. And we found a way to combine the gross appearance and microscopic findings by combining the true color 3D model based on block surface photographs and the accurate boundary of the solid tumor based on light microscopy in one model. Additionally, the model provided a chance to choose the most representative ROI for 3D exploration. Furthermore, it was proved that 3D models reduce the possibility of drawing inaccurate conclusions when analyses are only based on 2D pathological sections. Nevertheless, more data mining strategy should be developed for complete utilization of such a big data.

A lot of trouble emerged sequentially when researchers attempted extending from millimeter-sized specimens to the whole larynx (on the centimeter scale) Strategies to solve them might be helpful for future studies.

First, immunohistochemistry of whole-organ sections of human laryngeal cancer involves delicate handling, even though the laboratory techniques have been refined since Leroux-Robert^18^, first made sections of whole larynges in 1936. To date only Wittekindt et al.^19,20^ have reported techniques for the combination of whole-organ histology and immunohistochemistry in laryngeal cancer, by gentle decalcification and dehydration during paraffin embedding, and antigen retrieval. However, this procedure takes about 1 month. Here, we adopted Kawamoto’s film method^21–23^ to prepare whole-larynx frozen sections for both routine HE staining and immunohistochemical staining without fixation, decalcification and antigen retrieval, which took about 2 days. This is equal to the time for routine section preparation and makes it possible to prepare thousands of serial whole-organ sections for 3D reconstruction. The method can be used with different tissues of varying size, ranging from bones to soft tissue, though it has some obvious drawbacks. It requires skillful handling and occasional artifacts in the form of ice crystals and air bubbles may appear.

Second, panoramic digital microscopic images of the whole larynx at a resolution of 0.47 μm/pixel were obtained successfully using a customized whole-slide imaging system for large-scale sections, KF-PRO-005. This system provides accurate stitching results automatically. The size of the primary document can range from 2 to 4 GB and the scanning time is less than 10 min for each slice at a magnification of 400 ×.

Third, although substantial progress has been made in the development of rigid or non-rigid automatic registration algorithms for medical images in recent years^24,25^ and most medical imaging software include multiple models, many registration problems remain unresolved. For terabyte-scale serial section image stacks, both manual alignment and automated microscopy image registration are more challenging, compared with common clinical medical images like CT, MRI or colocalized PETCT scans that merge CT scans with nuclear medicine scans. In this study, the markers in the adhesive film copied from the holes in the positioning plastic square lid were effective for rough alignment. Additionally, block surface photographs were also used reliably, as an optional reference dataset for registration. As the requirement of accuracy of alignment increased with magnification, we developed corresponding registration strategies for different goals. After rough alignment, we failed to achieve automatic fine alignment in the whole large-image dataset using available software and corresponding modules due to the big dataset size and excessive time consumption. Here, we chose 100 panoramic images(100 ×)including ROI for accurate alignment combining manual and automatic methods. Manual alignment is not the best but remains an important and indispensable method. Special automatic image registration methodologies for terabyte-sized datasets should be developed in the future.

Finally, similar to the registration strategies, manual segmentation is indispensable in addition to automatic segmentation for some high-accuracy applications, such as confirming tumor budding. A program to identify the tumor boundary automatically is ideal rather than doing it manually, as it avoids some errors or deviations that may occur during manual processing. We have tried some segmentation modules in available 3D reconstruction software, which use several properties to group pixels or voxels, including intensity, intensity gradients, color, shape, orientation, and connectivity. However, they did not work well. As Clendenon et al.^26^ pointed that “Automatic segmentation is the ideal, but tracing the boundaries of regions by hand is still commonly done, due to the complexity of many biological structures.” Current technology is inadequate to solve the problem perfectly. Hence, automatic boundary identification tools based on block surface photographs or light microscopic images should be developed for future study. According to our experience, the magnetic lasso, as a semi-automatic edge detection tool is a powerful, yet underutilized tool in medical image segmentation.

Frankly, the whole process from preparing whole-organ slides to image processing and data analysis remains laborious, time-consuming, and expensive. For this reason, the dataset was obtained from a single case with supraglottic carcinoma of the larynx, in the current study. One model cannot represent all the characteristics of tumors at different stages. Hence, the feasibility in the clinical practice remains unclear. However, describing the characteristics of solid tumor in a 3D context with high resolution is a requirement by tumor’s intrinsic property. The technique used here is not just a research tool for oncology; it will be useful in routine procedure following advances in automatic tissue preparation, sectioning, staining, and automatic image processing in the future.

The choice of resolution of Z-axis is complicated. On one hand, compared to high resolution of X,Y-axis (0.47 μm), the resolution of Z-axis 10 μm is too long for reconstruction. Generally, the resolution in the Z-axis is the same as the section thickness for conventional light microscopic 3D reconstruction, which counteracts the advantages of improved resolution in the X–Y plane and reduces 3D isotropic resolution. In contrast, it did not show any meaningful high-resolution images in Z-axis, to help recognize pathological details from the macro perspective. In practice, we identified the boundary of tumor every 100 sections, which did not significantly affect the quality of reconstruction at macroscopic level. For other purposes, it may not be necessary to cut the specimen into 10-μm thickness serially and fewer sections can be collected thereby saving 3D reconstruction time. Although the labor cost in our study is massive, the preliminary data of our study showed the significance and necessity of accurate identification of tumor boundary based on whole organ sections.

In summary, it is feasible to establish a 3D light microscopy reconstruction of whole solid tumors at centimeter level by optimizing multiple currently available techniques. Although the technology is far from perfect, it may play a greater role in both oncology research and clinical practice in the future.

## Materials and methods

**Figure 3:**
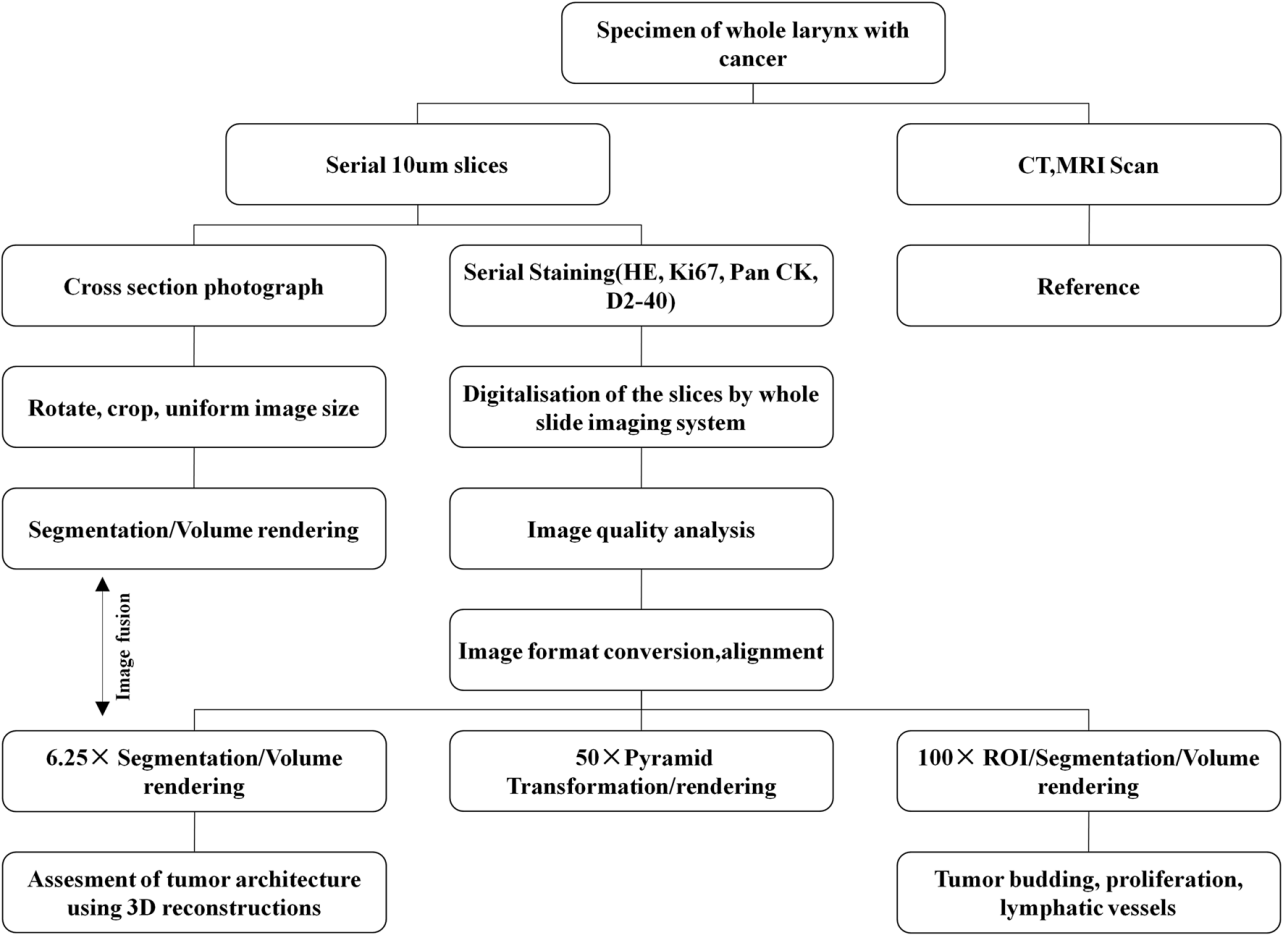
A diagram representing the flow of histological data processing and three-dimensional reconstruction of laryngeal cancer used in the study.

This study was reviewed and approved by the Scientific Research Ethics Review Committee of Shanxi Medical University (permission number: 2016LL128).The research was performed in accordance with the Declaration of Helsinki and informed consent was obtained from the patient. A diagram representing the flow of histological data processing and 3D reconstruction of tumor architecture used in this study is presented in Fig. 3.

### Patient and specimen

**Figure 4:**
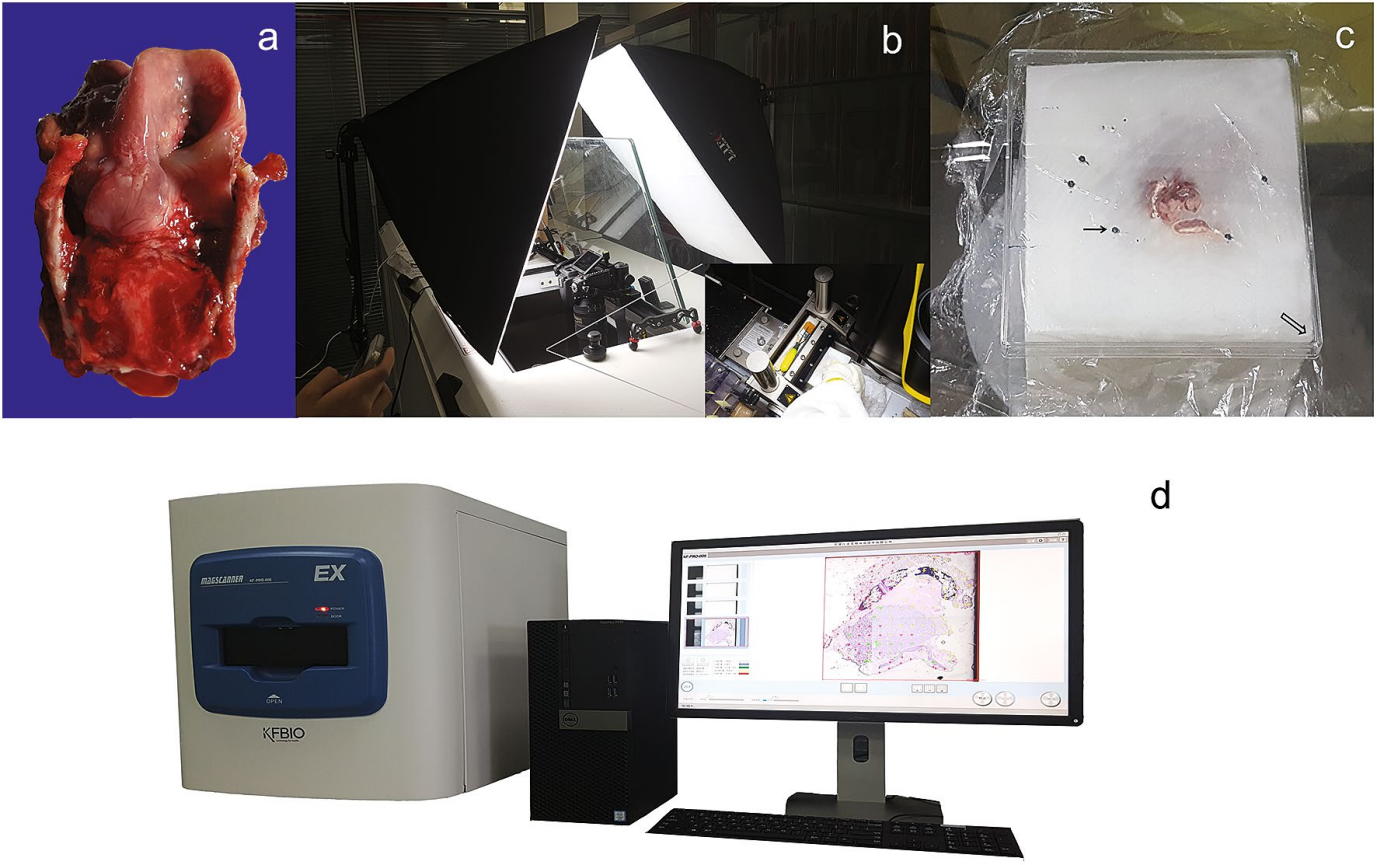
Specimen and equipment used in preparation of whole-organ frozen sections. (**a**) Gross appearance of the specimen was captured after dissection; (**b**) A fully computerized cryomacrotome (Leica CM3600 XP) and photographic system; (**c**) The plastic positioning plate for drilling fine holes (Black fine arrow) directly on the cryofilm, two adjacent edges of which were put around one corner (Black hollow arrow) of the block tightly; (**d**) Customized whole-slide imaging system KF-PRO-005 and operation interface.

A total laryngectomy specimen from a 70-year-old male patient with supraglottic laryngeal cancer (T3N0M0) was collected at the First Hospital of Shanxi Medical University on April 20, 2016. Laryngoscopic examination showed a mucosal eminence lesion on the laryngeal surface of the epiglottis with the left vocal cord fixed. A routine preoperative CT scan revealed that the lesion had invaded the epiglottis, pre-epiglottis space, and left ventricular bands. The gross appearance of the specimen was photographed after dissection (Fig. 4a). Immediately after excision, the specimen was temporarily stored in a portable icebox and then subjected to medical scanning as soon as possible. The whole organ was placed in a position similar to its arrangement in the human body. High-resolution CT scan and MRI (3.0-T) were performed as reference data to help find any obvious mistake during alignment.

### Preparation of whole-organ frozen sections from unfixed and undecalcified laryngeal tissues using Kawamoto’s film method

Kawamoto’s film method^21–23^ was used to prepare frozen sections of the whole larynx for both immunohistochemical staining and routine HE staining, without fixation and decalcification. The method was slightly modified to fit the sections of the whole larynx. The embedding medium (SCEM), a low-temperature resistant adhesive film (Cryofilm 2C12), and the mounting medium (SCMM) were obtained from SECTION-LAB Co., LTD. (Hiroshima, Japan). The cryofilm was cropped to 4.5 × 9.5 cm. The procedures and materials used are described below.

#### Freeze embedding and section preparation

The sample was quickly frozen in hexane dry ice, and then put in a customized stainless-steel container (inner diameter 10 × 10 × 7 cm) with precooled embedding medium, in an upright position. A fully computerized cryomacrotome (Leica CM3600 XP; Leica Biosystems Inc., Buffalo Grove, USA, Fig. 4b) and a disposable tungsten carbide blade (H45L; SECTION-LAB Co., LTD. Hiroshima, Japan) were used for sectioning. The thickness was set to 10 µm and automatic mode of sectioning was chosen with a sledge speed of 8 mm/s. Prior to removing a section, a piece of cryofilm was attached to the specimen block surface using a brush. After the completion of sectioning, the cryofilm was fixed to a customized glass slide (5 × 7 cm; Shanghai Jinan Biological Co., LTD. China) with the specimen side up, using a custom-made plastic basket for subsequent staining.

#### Artificial fiducial markers for registration

To ensure the accuracy of registration, artificial fiducial markers were made on the cryofilm using a special plastic positioning plate (Fig. 4c). First, two sides of one right angle of the square plastic lid were tightly placed around the corresponding corner of the block. Four fixed fiducial holes just around the whole larynx were drilled through the square plastic lid as a template. Then, after pressing the cryofilm onto the block surface to cover the entire sample, corresponding marker holes were punched in each cryofilm by a fine needle through the reference holes in the lid vertically. The diameter of the hole was 0.5 mm. Although the absolute position of the holes marked in the cryofilm was different each time, the position of the hole relative to the whole block surface and the larynx did not change, enabling its use as a kind of registration marker.

#### Sectioned block surfaces photography

During the intervals between sectioning, block surface images were captured by a photographic system, consisting of a NikonD750 camera (24 million pixels; Nikon Imaging (China) Sales Co., Ltd. China) with an AF-S Nickel 28–300 mm f/3.5 ~ 5.6 GEDVR lens, two 60 W LED soft light boxes (50 × 70 cm) for improving luminous intensity and brightness, and a cell phone acting as a wireless monitor and controller (Fig. 4b). The lens of the camera was mounted perpendicularly just above the center of the block surface using the slide rail, which was parallel to the block surface.

#### HE and immunohistochemical staining of sections

The sections were successively stained with HE and immunostained with antibodies AE1/AE3, Ki-67, and D2-40 (Zhongshan Jinqiao Biotechnology Co., LTD. China), which are commonly used molecular markers for marked squamous cells, proliferating cells, and lymphatic cells, respectively. All procedures were performed in accordance with the recommended protocols of Kawamoto’s film method. Phosphate buffered saline (PBS) without primary antibodies was used as a blank control. Normal epithelium in the sections was used as a positive control for cytokeratin. Tonsils served as positive controls for D2-40.

### Microscopic scanning and digitalization of whole slide

KF-PRO-005 (Konfoong Biotech International Co., LTD. Ningbo, China) (Fig. 4d), a customized whole slide imaging system, was used to scan the whole laryngeal section at a resolution of 0.5 μm/pixel. The hardware included a 3CCD linear camera, plan apochromatic objective (20 ×, 0.75 N.A.; Olympus Corporation, Japan), linear magnetic shaft motor (positional accuracy: 50 nm), and high-precision grating ruler, that can increase the quantity of image stitching and ensure repeat positioning accuracy when scanning large samples. Supporting software included K scanner, K viewer, and a batch processing program for image format conversion from Kfb to TIFF. The software was specially optimized for large sections in terms of the stitching algorithm, data acquisition speed and data compression.

Lenovo Thinkstation P700 (2 × Intel Xeon CPU E5-2630V3@2.4 GHz, 128 GB DDR4 RAM, NVIDIA Quadro M4000; Lenovo, Lenovo group Co.,LTD. Beijing, China) was used for image processing. First, the raw data were imported into Photoshop CC (PS CC, Adobe Systems Inc, San Jose, CA, USA) and the image was cropped along the border of the block surface after rotating and translating using magic lasso tool. Then, all images were adjusted to the same size (10 × 10 cm) and resolution (2455 × 2455 pixels, 245.5 pixels/cm; the lowest resolution of all images defined by the resolution of the last image at the bottom of the block) and were saved as a TIFF file without compression. The size of the whole block surface remained nearly unchanged during the slicing; therefore, the contour of the block surface could be used to make all the captured images uniform and roughly aligned. Next, a plain black background replaced the region of embedding medium and a few images with different brightness were adjusted. Finally, the roughly aligned dataset was imported into Thermo Scientific Amira 6.0 software (Thermo Fisher Scientific and Roper Technologies Inc, OR, USA), and the alignment was further improved using the automatic optimization method via the least-squares alignment mode.

### Preprocessing and image registration of light microscopic images

First, the original image format Kfb was converted to TIFF at different magnification rates (6.25 ×, 50 ×, 100 ×) using a batch processing program. The microscopic images at 6.25 × were introduced into Photoshop CC and put on a 10 × 10 cm background plate with the same resolution, in which the positioning marker had already been copied from the fiduciary holes in the positioning lid. Then, the pathological image was manually moved to the original position in the block by putting the fiducial holes in the cryofilm on the marker in the background. After rough alignment, an automatic optimization method via the least-squares alignment mode in Amira was used to test and improve the quality of the alignment. Finally, to enhance the visual effects of 3D reconstruction, the region outside the tissues was replaced by a plain black background. The preliminary processing of 50 × images was performed in the same manner as the 6.25 × ones, where the aligned 6.25 × slices could act as a reference for the alignment of 50 × images. Then, the images were precisely aligned manually according to the anatomical landmarks at high power. Anatomical markers, such as laminae of thyroid cartilage act as important reference points to evaluate the accuracy of the reconstruction.

A different strategy was used for the alignment of 100 × images, since the size of a single TIFF file of 100 × images was too large for the conventional image processing software. First, the target raw image data (about 2 GB) were imported in a new canvas (10 × 10 cm) in Photoshop CC without loss of pixels, followed by alignment according to the correspondingly aligned 6.25 × image. Then, the area of interest (ROI) of 0.4 × 0.4 cm at the same position of each whole slide was cropped and saved as a new TIFF file according to the coordinates. Thereafter, the cropped sections were further aligned by manual and automatic methods in Amira.

### Segmentation of anatomical structures and molecular marked cells

The boundary of the tumor in the cross-sectional photographs and in the light microscopic sections at 6.25 × was identified and delineated using magnetic lasso tool in Photoshop CC, which is an edge detection tool and can be used to create semi-automatic outline selections. The location of the sensing circle was determined by the operator’s approximate identification of area of tumor margin. The same contrast and frequency setting guarantee automatic margin detection and selection of the pixels around them. Suspicious pathological structures were confirmed in higher power light microscopy digitized images. This gave it a high degree of precision than an automatic segmentation algorithm. The hyoid bone, thyroid cartilage, epiglottis cartilage, arytenoid cartilage, and cricoid cartilage were identified and labeled in block surface images using the wand tool.

In the ROI, laryngeal carcinoma nests (marked by pan-CK), cell proliferation (marked by Ki-67), and intratumoral and peritumoral lymph vessels (marked by D2-40) were automatically identified and labeled in Image Pro Plus 6.0 (Media Cybernetics Inc, Bethesda, USA) using color cube based segmentation model. Tumor budding, as a single tumor cell or a cell cluster of up to four tumor cells at the invasive front and within the tumor body, was confirmed again after preliminary automatic segmentation, in accordance with the definition of colorectal cancer recommended by the International Tumor Budding Consensus Conference^27^. Next, the isolated markers were imported into Amira and readily segmented using a thresholding method for volume rendering. The different datasets were introduced into Amira simultaneously for fusion image analysis. The uniform size of multimodality images enabled fusion directly without another procedure of alignment.

### 3D imaging and visualization

Amira was used in smaller datasets for its high-performance 3D visualization and multiple object management. To achieve better rendering of complex spatial structures, the whole dataset was reconstructed using isosurface rendering or volume rendering with or without segmentation. Vaa3D, an open-source 3D software, was used for 3D reconstruction and exploration of large-scale microscopic images. Compared with any other software, it better facilitates the 3D visualization of terabyte-sized stacks in real time on an ordinary desktop computer^28–30^. The 50 × series of 2D TIFF files was converted and stored in a hierarchical data structure, first by the TeraConverter module in Vaa3D, where each hierarchical level represents a different resolution that can be independently loaded upon request that enables rapid access to small parts of an image volume at different scales. Then, the new exported datasets were opened in Terafly on the platform of the Vaa3D system to achieve a real-time response for true 3D rendering (real-time maximum intensity projection or alpha blending).

Different modes of 3D software were tried for rendering volumetric image, including maximum intensity projection (MIP), minimum intensity projection (mIP), average-intensity projection, alpha-blending projection, and shaded or classical texture-based volume rendering. The final choice of the 3D image was the one that best helped in understanding the image content.

## Supplementary information

Supplementary Information 1.

Supplementary Information 2.

Supplementary Video 1.

Supplementary Video 2.

Supplementary Video 3.

Supplementary Video 4.

Supplementary Video 5.

Supplementary Video 6.

Supplementary Video 7.
