# Supplementary material for: Three-dimensional reconstruction of laryngeal cancer with whole organ serial immunohistochemical sections
Source: Sci Rep. 2020 Nov 3;10:18962. doi: 10.1038/s41598-020-76081-7 (PMC7642254; doi:10.1038/s41598-020-76081-7)
Supplement: Supplementary file 1 — Supplementary Information 1. [file 41598_2020_76081_MOESM1_ESM.pdf]

Supplementary material

Three-dimensional Reconstruction of Laryngeal Cancer with Whole Organ Serial Immunohistochemical Sections

Tian Jun[1]; Qian Bo[2]; Zhang Sanmei[3]; Guo Rui[1]; Zhang Hui[4];J-P Jeannon[5]; Jin Rongxiu[6]; Feng Xiang[7];Zhan Yangni[7];Liu Jie[7]; He Pengfei[7];Guo Jue[7] ; Li Le[8]; Jia Yue[7]; Huang Fuhui[7];Wang Binquan[7]*

VIDEO 1 3D model of tumor bulk and framework of larynx without segmentation based on block surface image data.

VIDEO 2 The relatively accurate outline of the tumor bulk based on pathology was mapped to the 3D model based on block surface image data without segmentation of laryngeal frameworks.

VIDEO 3 The relatively accurate outline of the tumor bulk based on pathology was mapped to the 3D model based on block surface image data with segmentation of laryngeal frameworks.

VIDEO 4 The relationship between the tumor bulk and the arytenoid joint.

VIDEO 5 Tumor budding in round-shaped tumor aggregates around the ventricle.

VIDEO 6 Tumor budding in palisading-shaped tumor aggregates around the ventricle.

VIDEO 7 Exploration of whole-mount sections of human laryngeal cancer at different scales using the Terafly module in Vaa3D.
