# Supplementary material for: Three-dimensional reconstruction of laryngeal cancer with whole organ serial immunohistochemical sections
Source: Sci Rep. 2020 Nov 3;10:18962. doi: 10.1038/s41598-020-76081-7 (PMC7642254; doi:10.1038/s41598-020-76081-7)
Supplement: Supplementary file 2 — Supplementary Information 2. [file 41598_2020_76081_MOESM2_ESM.docx]

**TITLEPAGE**

Three-dimensional Reconstruction of Laryngeal Cancer with Whole Organ Serial Immunohistochemical Sections

*Tian Jun^1^; Qian Bo^2^; Zhang Sanmei^3^; Guo Rui^1^;* *Zhang Hui^4^;J-P Jeannon^5^; Jin Rongxiu^6^; Feng Xiang^7^;Zhan Yangni^7^;Liu Jie^7^; He Pengfei^7^;Guo Jue^7^; Li Le^8^; Jia Yue^7^; Huang Fuhui^7^;**Wang Binquan^7*^*

*1.**Department of Otolaryngology, Head & Neck Surgery,* *Beijing Friendship Hospital, Capital Medical University. Beijing City, China*

*2.Department of General Surgery, The General Hospital Of Taiyuan Iron&Steel Company, Taiyuan City, China*

*3.Medical Department of Medical Insurance, Beijing Friendship Hospital, Capital Medical University, Beijing City, China*

*4.Imaging Department, Shanxi Medical University, Taiyuan City, China*

*5.Department of Otolaryngology, Head & Neck Surgery, Guy's & St Thomas NHS Hospital, London, United Kingdom*

*6.Department of Nursing, Peking University Shenzhen Hospital, Shenzhen City, China*

*Department of Otolaryngology, Head & Neck Surgery, the First Hospital of Shanxi Medical University, Taiyuan City-030001, China*

*8. Department of Head and Neck, Shanxi Provincial Cancer Hospital, Taiyuan City, China*

Running title: 3D Reconstruction of Whole Laryngeal Cancer

Keywords: Whole-Organ Sections; Three-Dimensional Reconstruction; Light Microscopy; Laryngeal cancer

Corresponding author: Binquan Wang, wbq_xylc@126.com, No. 85, Jiefang South Road,Taiyuan City-030001 China.
